# Impact of Ethanol Infusion to the Vein of Marshall in Atrial Fibrillation and Atrial Tachycardia

**DOI:** 10.3390/jcdd11070183

**Published:** 2024-06-21

**Authors:** Masateru Takigawa, Shinsuke Miyazaki, Tetsuo Sasano

**Affiliations:** 1 Department of Cardiovascular Medicine, Tokyo Medical and Dental University, Tokyo 113-8510, Japan; 2 Department of Advanced Arrhythmia Research, Tokyo Medical and Dental University, Tokyo 113-8510, Japan

**Keywords:** ethanol infusion, ligament of Marshall, vein of Marshall, atrial fibrillation, atrial tachycardia

## Abstract

The ligament of Marshall is an epicardial structure characterized by its composition of fat, fibrous tissue, blood vessels, muscle bundles, nerve fibers, and ganglia. Its intricate network forms muscular connections with the coronary sinus and left atrium, alongside adjacent autonomic nerves and ganglion cells. This complexity plays a pivotal role in initiating focal electrical activities and sustaining micro- and macro-reentrant circuits, thereby contributing to the onset of atrial fibrillation and atrial tachycardia. However, endocardial ablation in this area may encounter challenges due to anatomical variations and insulation by fibrofatty tissue. Combining ethanol infusion into the vein of Marshall with radiofrequency ablation presents a promising strategy for effectively and safely eliminating this arrhythmogenic structure and terminating associated tachycardias.

## 1. Introduction

In 1850 [1], John Marshall made a groundbreaking discovery of a unique ligamentous structure hidden at the epicardial lateral left atrium (LA), positioned along the ridge between the left atrial appendage (LAA) and the inferior and superior left pulmonary veins (PVs). This intricate formation, consisting of pericardium, fibrous tissue, fat, blood vessels, and nerves, was eventually named the ligament of Marshall (LOM).

**Figure 1:**
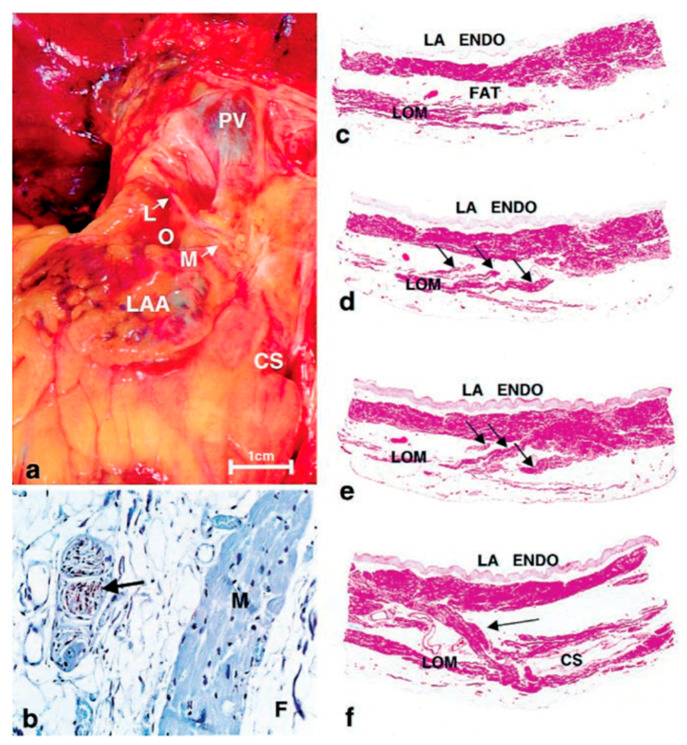
Histology of ligament of Marshall. (**a**) Photo showing location on the posterior surface of heart. (**b**) Immunohistochemical staining for tyrosine hydroxylase showing positively in nerve (brown staining—arrow). M, myocardium; F, fat (avidin–biotin–peroxidase, ×120). (**c**–**e**) Subserial sections showing the LOM, isolated from the left atrial wall (in **c**), with 3 tracts (arrows) emerging from it (in (**d**)) and eventually entering the atrial wall (in (**e**)) (hematoxylin and eosin [H&E] stain ×10). (**f**) Section from the lower end of LOM showing tract entering the left atrial wall (arrow) and CS (H&E stain ×10). CS, coronary sinus; LAA, left atrial appendage; LOM, ligament of Marshall; PV, pulmonary vein; LA ENDO, left atrial endocardium. This figure is reproduced from the original manuscript [2] with permission from Elsevier.

In 2000 [2], Kim DT and colleagues examined seven postmortem human hearts to demonstrate the gross anatomical and microscopic features of the LOM in detail. Their thorough investigation elucidated that the LOM in humans, innervated by sympathetic nerve fibers, is significantly more complex than in canines. It features intricate ganglia, numerous sympathetic nerve fibers, abundant myocardial bundles, and vasculature, and displays multiple myocardial tract insertions into the coronary sinus (CS) and the LA free wall, all encased in fibro-fatty tissue insulation. These profound insights suggest the potential risk of arrhythmogenicity of the LOM, and the difficulty in eliminating these substrates by endocardial ablation alone (see Figure 1a) [2].

In recent times, the significance of the LOM in atrial fibrillation (AF) [3,4,5,6,7,8] and scar-related atrial tachycardia (AT) [9,10,11,12,13] has surged in importance. Especially in complex atrial tachyarrhythmias, the LOM may play a critical part in the maintenance of these arrhythmias, and abolishing the LOM holds promise as a viable adjunctive therapy. However, achieving complete transmural ablation of this epicardial structure solely from the endocardial approach presents challenges. Ethanol infusion into the vein of Marshall (EI-VOM) emerges as a promising complementary strategy to create a lasting lesion in this area [14,15,16,17,18,19,20,21,22,23,24,25,26].

In this extensive review, we examine the anatomy and electrophysiological characteristics of the LOM, discuss its role in atrial arrhythmias, and investigate effective methods for targeting this arrhythmogenic substrate through radiofrequency ablation and ethanol injection into the vein of Marshall (EI-VOM).

## 2. Anatomy of the LOM

**Figure 2:**
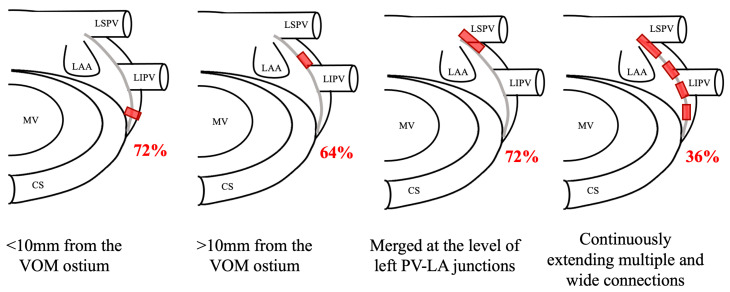
Various connections between the LA and LOM. Locations of connections between LA and LOM is illustrated in red squares. The incidence of each connection is described in red characters. CS, coronary sinus; LA, left atrium; LAA, left atrial appendage; LIPV, left inferior pulmonary vein; LSPV, left superior pulmonary vein; MV, mitral valve.

The LOM consists of residual embryonic structures derived from the sinus venosus and the left cardinal vein. This complex structure includes muscle bundles, blood vessels, adipose tissue, fibrous structures, ganglia, and nerve fibers [1,2,27]. Anatomically, the LOM can be divided into proximal, mid, and distal segments [28]. Research conducted by Makino M et al. [27] analyzed the distribution of these components in 25 postmortem human hearts, which comprised six cases with AF and nineteen without (Figure 2). The investigation revealed the presence of close connections at the CS junction in 18 instances, demonstrating a prevalence of 63% among controls and 100% in AF cases. Furthermore, distant connections were documented in 16 cases, occurring in 63% of controls and 67% of AF cases. Connections at the LPV–LA junction were also observed in 18 cases, with a frequency of 68% in controls and 83% in AF cases. In addition to these localized connections, the study identified extensive and multiple wide connections in nine cases, affecting 31% of controls and 50% of AF cases. This detailed examination underscores the complex and variable anatomy of the LOM and its potential relevance in cardiac structural and electrophysiological abnormalities. 

The neural composition of the LOM [7,29] is a critical aspect of its anatomical and functional identity. Immunohistochemical studies have revealed that the LOM is extensively innervated by autonomic nerves and contains numerous ganglia. Makino M et al. [27] demonstrated a dense concentration of sympathetic nerve fibers around the PV–LA junctions, while parasympathetic ganglia are predominantly located at the CS juncture. Notably, there is a gradient observed in the distribution of these neural elements along the LOM: sympathetic nerve fibers diminish from the distal to the proximal segments, whereas parasympathetic ganglia exhibit an increase. This detailed mapping underscores the complex interplay of autonomic innervation within the LOM, suggesting its potential influence on cardiac function and arrhythmogenesis.

The vein of Marshall (VOM), a prominent vascular structure within the LA, is encapsulated by the LOM. Serving as a critical drainage pathway, the VOM collects blood from the posterior and posterolateral walls of the LA, descending obliquely and inferiorly toward the CS. Its entrance is typically located just proximal to the Vieussens valve within the proximal segment of the CS. Notably, coronary sinus venography reveals the presence of this vein in over 90% of examined cases [30].

## 3. Relation between LOM and AF

**Figure 3:**
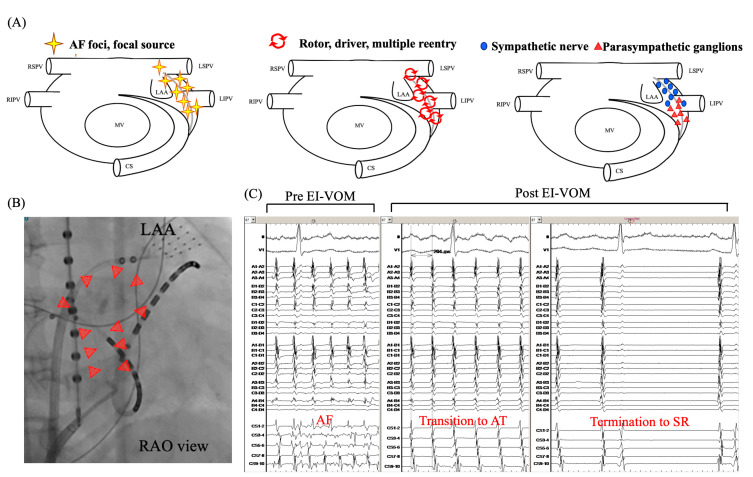
**Underlying mechanisms maintaining AF related to the LOM.** Several mechanisms initiating or maintaining AF related to the LOM are depicted (**A**). When the LOM is responsible for the AF initiation or perpetuation, EI-VOM may effectively terminate AF (**B**,**C**). Red triangles in (**B**) show the distribution of staining after EI-VOM. (**A**) is modified from the original manuscript by Takigawa M et al. [36]. AF, atrial fibrillation; CS, coronary sinus; EI-VOM, ethanol infusion to the vein of Marshall; LAA, left atrial appendage; LIPV, left inferior pulmonary vein; LSPV, left superior pulmonary vein; MV, mitral valve; RAO, right anterior oblique; RIPV, right inferior pulmonary vein; RSPV, right superior pulmonary vein.

The LOM can be linked to AF in three key ways: it can act as a source of arrhythmia, serve as a substrate for its propagation, and be involved in autonomic innervation contributing to the development and persistence of AF (Figure 3). Scherlag BJ et al. first demonstrated in an experimental study that the LOM may be an arrhythmogenic source [31]. Multiple subsequent reports have shown ectopic beats from the LOM triggering paroxysmal AF [3,4,32,33]. Hwang C et al. [3] successfully demonstrated that AF initiation was observed from the muscle bundle within the LOM and ablating the insertion site of the VOM effectively terminated AF in four out of six patients. These findings suggest the potential arrhythmogenicity at the LOM as the source of focal AF. Liu CM et al. [34] investigated 254 patients with persistent AF, including 67 (26%) patients with non-PV foci, showing that 20 (19.6%) patients had a LOM origin. Lin WS et al. [35] examined 240 AF patients with a total of 358 ectopic foci. Seventy-three foci initiating AF were observed in sixty-eight (28%) patients, including six (8.2%) from the LOM. While AF more frequently originates from the distal region than the proximal region of the LOM, ectopic AT tends to arise more frequently from the proximal region of the LOM, particularly in locations near the CS [5].

The LOM also plays a crucial role in AF as an arrhythmogenic substrate. Studies have shown that left PV reconnection via epicardial connections through the VOM can occur in a minority of cases post-PV isolation. Additionally, research has revealed that patients with epicardial connections, particularly between the left PVs and the LOM, have lower acute success rates in pulmonary vein isolation and a higher risk of atrial tachyarrhythmia recurrence [37]. As mentioned above, Makino M et al. [27], demonstrated the various connections between the LA and the LOM. The LOM was a prevalent feature, present in 25 hearts, with remarkable connections near to (*n* = 18, 72%), and distant from (*n* = 16, 64%) the CS juncture connections (Figure 3). Connections between the left PV and LA were observed in 18 (72%) cases, further underscoring the complexity of this anatomical landscape. These findings were corroborated by Han S et al. [32], who electrophysiologically demonstrated these anatomical variations. Specifically, their investigation revealed diverse patterns of myocardial connections, including single connections between the LOM and CS muscle sleeves, dual connections to both the LA and CS along the left PVs, and multiple connections within the MB region. During sinus rhythm, activation patterns exhibited notable variability and irregularity, while AF consistently showed rapid and fractionated complex activations. These anatomical and electrophysiological insights collectively imply that the complex myocardial connections between the LOM and LA may significantly contribute to the development and persistence of reentrant circuits that drive AF [5,6,27,28]. Both in animals and humans [8,38], the shortest activation intervals and highest dominant frequencies at the LOM were observed in sustained AF, suggesting that the LOM region may lead to AF in some cases.

The LOM is also implicated in the genesis of AF as a source of arrhythmogenic autonomic innervation [2,7]. Yu X et al. [39] explored the effects of ablating the distal segment of the LOM on atrial electrical remodeling. To assess the changes, they conducted rapid atrial pacing on canine models, applying the procedure before and after ablation of the distal LOM. This study aimed to determine the impact of this ablation on atrial remodeling, as indicated by shorter atrial effective refractory periods (ERPs), greater ERP dispersion, and the ease of induction and persistence of AF. The authors concluded that ablating the distal LOM might reduce cardiac sympathetic activity, potentially lowering the inducibility and persistence of AF. Báez-Escudero J et al., [40] examined 80 patients out of 133 AF patients undergoing VOM ethanol infusion procedure, who were able to complete high-frequency stimulation from the VOM before and after the EI-VOM, in order to determine whether EI-VOM can eliminate intrinsic cardiac nerves responses such as atrioventricular (AV) nodal conduction slowing (asystole > 2 s or R-R interval prolongation > 50%) and AF induction. While AF induction and intrinsic cardiac nerve responses were evident before EI-VOM, these responses were effectively abolished post-procedure. These findings underscore the role of intrinsic cardiac nerve responses in AF and highlight the therapeutic impact of parasympathetic denervation through EI-VOM.

## 4. LOM-Related AT

The LOM is frequently associated with reentrant ATs following AF ablation. Vlachos et al. identified two distinct types of LOM-related reentrant ATs [9], namely LOM-related PMF and LOM-related localized AT, in 140 patients who had received at least one previous ablation procedure (mean: 2.9 ± 1.5) for persistent AF, either as a standalone procedure or combined with mapping and ablation of AT. The stepwise approach was performed in 91 (65%) patients who presented with left ATs after ablation for persistent AF, with 49 (35%) undergoing body surface mapping system-based ablation. Among 199 scar-related ATs in these patients, 30.2% were diagnosed as LOM-related, comprising 15.6% PMFs and 14.6% localized reentries. Another report [10] demonstrated that LOM-related PMF accounted for up to 11% of 38 patients with PMF with prior AF ablation (*n* = 4) or valve surgery (*n* = 2). Importantly, the mitral isthmus area was endocardially scarred or damaged, due to prior procedures or spontaneously. Following multiple procedures, residual epicardial connections associated with the LOM are more likely to contribute to the circuit of ATs. Our study tracked 147 patients with AF-ablation-related ATs, mapped using Rhythmia^TM^ (Boston Scientific, Marlborough, MA, USA) [11]. Of these, 44 patients underwent redo procedures, allowing for a comparison between the AT maps during the redo procedures and the original AT circuit, demonstrating the mechanism of AT recurrence. When the initial AT involves PMF or roof-dependent macroreentrant AT, the recurrence mirrors the original circuit in 57.7% and 44.4% of cases, respectively. Notably, 51.2% of recurrent ATs include epicardial structures in their circuits, which is particularly remarkable for PMF, where the CS/VOM system plays a role in 75% of redo cases. In contrast, when the initial AT is either focal AT, CTI-dependent AT, or non-anatomical macroreentrant AT, the subsequent recurrence tends to form circuits distinct from the original ones. Our results align with previous studies highlighting the difficulty in achieving transmural linear lesions across the mitral isthmus and the LOM [41]. Anatomical and histological evidence shows that the LOM has multiple connections to the LA and is insulated by fatty tissue [2], indicating that it could serve as a substrate for ATs.

**Figure 4:**
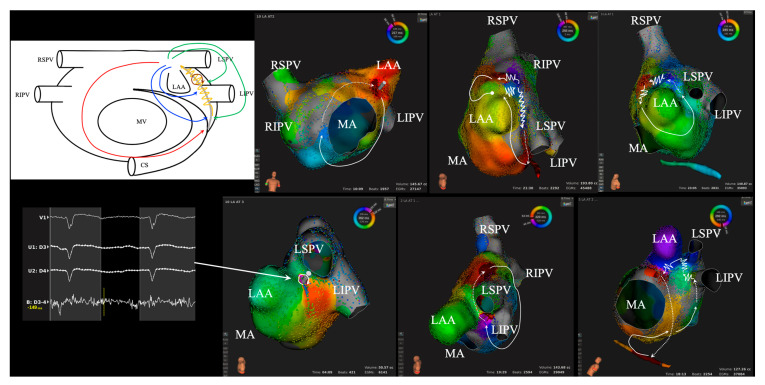
**Multiple complex AT circuits using the LOM displaying centrifugal activation.** AT associated with the LOM should be considered whenever centrifugal activation is detected at sites along the LOM in the lateral LA. The endocardial insertion points from epicardial structures often manifest as centrifugal activation, which must be distinguished from true focal activation—an atypical occurrence in this region (endocardial lateral LA). AT, atrial tachycardia; CS, coronary sinus; LAA, left atrial appendage; LIPV, left inferior pulmonary vein; LSPV, left superior pulmonary vein; MV, mitral valve; RIPV, right inferior pulmonary vein; RSPV, right superior pulmonary vein.

In many cases, various arrhythmogenic circuits—including macro-reentry and micro-reentry around the LOM are observed, depending on prior procedures. These circuits may be intricate, complicating the identification of the precise mechanism of arrhythmias. Recognizing LOM-related ATs is crucial when arrhythmias reoccur after extensive ablation. The detection of centrifugal activation patterns from specific endocardial regions along the lateral left atrium, corresponding with the LOM distribution, is facilitated by 3D mapping systems. This approach helps to differentiate between reentrant (pseudo-focal) ATs involving the CS and LOM structures, and true focal ATs [12,13] (Figure 4). 

## 5. Impact of EI-VOM on AF Outcome

Considering the role of the LOM as both a focus and substrate for AF, alongside the challenges in eliminating this epicardial structure through endocardial ablation, EI-VOM emerges as a necessary alternative to effectively eradicate this structure. A recent prospective, multicenter, randomized controlled trial, the VENUS-AF trial [42], revealed the remarkable superiority of combining EI-VOM with conventional catheter ablation (CA) alone in significantly reducing AT/AF recurrence and burden compared to CA alone. This groundbreaking study encompassed 343 patients suffering from symptomatic persistent AF, comparing those who underwent CA alone (*n* = 158) to those who received EI-VOM in addition to CA (*n* = 185). Despite 30 patients (16.2%) in the EI-VOM plus CA group not achieving successful EI-VOM, there were still significantly fewer recurrences observed after a single procedure, and the discontinuation of antiarrhythmic drugs in the EI-VOM plus CA group (49.2%) compared to the CA alone group (38%). Notably, a sub-analysis of the VENUS trial unveiled that the procedural outcome significantly improved when mitral isthmus block was accomplished [43].

While the evidence regarding the impact of adjuvant EI-VOM on AF’s clinical outcome has not been robustly established by several multiple prospective randomized studies to date, several meta-analyses have been presented [44,45,46,47]. He Z et al. synthesized a total of 10 studies [20,21,26,33,34,42,48,49,50,51] involving 1322 patients, of which five studies (923 patients) investigated the efficacy of EI-VOM combined with CA versus CA alone in AF patients [45]. Despite the analysis not reaching significance in terms of the recurrent rate of AF and/or AT between the EI-VOM combined with CA group and CA alone group (RR: 0.68, 95% CI: 0.45–1.02, *p* = 0.06), focusing on the persistent AF population [33,34,42] revealed a significantly lower recurrence of AF and/or AT in the EI-VOM combined with CA group compared to the CA alone group (RR: 0.58, 95% CI: 0.35–0.96, *p* = 0.04). In a meta-analysis by Ge W et al. [46], a comprehensive review of nine studies [15,20,22,25,34,42,48,52,53], encompassing a staggering 2508 patients experiencing AF, was meticulously undertaken. Among this cohort, 1028 patients underwent EI-VOM + CA, while 1605 underwent CA alone. Despite this diverse population, inclusive of not only persistent AF but also paroxysmal AF and PMF, the profound efficacy of EI-VOM in mitigating the recurrence of atrial arrhythmias was unmistakable (RR = 0.70 [0.53–0.91], I^2^ = 81%). Upon conducting subgroup analyses, comprising five studies [20,22,34,42,52] primarily focusing on persistent AF or non-paroxysmal AF and two studies [15,25] solely dedicated to PMF, the impact of EI-VOM + CA was even more pronounced (0.63 [0.42–0.95] and 0.52 [0.35–0.77], respectively).

Derval N et al. [51] explored the impact of EI-VOM in a single-center study, known as the Marshall-PLAN. In this study, a novel comprehensive anatomical ablation strategy including Marshall bundle elimination, pulmonary vein isolation, and line completion for anatomical ablation [Marshall-PLAN]) was examined. In addition to the elimination of the Marshall bundle using EI-VOM and CA along the endocardial ridge and epicardial muscular bundles at the CS great cardiac vein, the PVs were isolated, followed by the complete linear lesions to the roof, mitral isthmus, and CTI. Seventy-five patients with persistent AF were involved in the study, and EI-VOM was achieved in 92%. Finally, the full Marshall-PLAN strategy was completed in 91%. After a year, 72% of patients were free from AF/AT without antiarrhythmic drugs, rising to 79% in those who received the complete Marshall-PLAN. They are now conducting a multicenter study comparing Marshall-PLAN to PVI. Additionally, Lai Y et al. [52] compared a ‘2C3L’ approach including bilateral PVIs and three anatomical lines on the mitral isthmus, roof, and CTI, performed solely by RF ablation with an upgraded ‘2C3L’ approach in which EI-VOM was performed in addition to the RF ablation for the same approach to treat persistent AF [52], finding better outcomes at the 12-month follow-up. A randomized trial named ‘PROMPT-AF’ is currently ongoing to further evaluate these approaches [54].

In contrast, the MARS trial, a prospective multicenter randomized controlled study, initially reported conflicting results. This trial involved 80 persistent AF patients undergoing redo-CA. Participants were randomly assigned to either CA alone or CA combined with EI-VOM. While EI-VOM achieved a remarkable 90% success rate, there was no significant difference between the two groups in achieving an MI block or in maintaining freedom from AF/AT 12 months post-procedure [55]. The clinical impact of EI-VOM may fluctuate based on the study population and the complementary ablation strategies employed. Therefore, a meticulous evaluation of long-term outcomes is imperative.

## 6. Reliable Efficiency and Safety in MI Ablation

Achieving a complete MI block using RF presents a considerable challenge, with reported success rates ranging from 31% to 92% [56,57,58,59,60,61]. Often, RF applications within the CS are required, ranging from 59% to 91%. Anatomical variations of the MI, such as its length and thickness, along with complex structural relationships between the MI and neighboring structures like the CS/VOM and circumflex artery, may contribute to this challenge [62,63,64]. Additionally, the distribution of fatty tissues insulating the VOM [2] on the epicardial side can impact RF conductivity. 

In addition, assessing the achievement of a complete mitral isthmus (MI) block poses challenges. A pseudo-MI block, where conventional pacing shows a complete MI block but mapping reveals an incomplete block, occurs in about 20–30% of cases [65,66,67,68]. This misdiagnosis often results from residual epicardial conduction despite an endocardial block [11,68]. To address this, different linear lines like anterior or anteroseptal lines are sometimes chosen [69,70,71], but they can increase the risk of bi-atrial tachycardias [72,73,74], which are hard to treat. Ideally, the posterior MI region, bridging the lateral mitral annulus to the left pulmonary veins, is the optimal site for achieving a robust endocardial-to-epicardial block. This strategic area, being the last to activate during SR, allows for the establishment of a bi-directional block without disrupting the natural conduction of the LA during SR. However, the pursuit of creating durable lesions in this area by increasing RF power, extending RF duration, or enhancing contact force significantly heightens the risk of steam pops. 

EI-VOM has emerged as a supplementary method with RF to disrupt epicardial conduction [11,16,18,20,25,42]. This combined approach boasts an exceptionally high success rate, ranging from 98% to 100%, in achieving a mitral isthmus block [20,25,75,76]. The most recent randomized controlled study comparing EI-VOM patients (*n* = 45) and conventional RF patients (*n* = 44) demonstrated that EI-VOM can reduce acute reconnection after an MI bidirectional block and significantly increase first-pass MI blocks compared to conventional RF [77]. Further, a recent meta-analysis delved into the impact of EI-VOM on MI ablation, pooling data from six studies [15,20,22,25,42,52]. Among 660 patients who underwent EI-VOM, 624 (94.5%) achieved an MI block, a significant improvement compared to the 739 (82.7%) of 964 patients who underwent CA alone (RR = 1.29 [1.11–1.50], I^2^ = 93%). The sub-analysis of another meta-analysis including two studies [20,42] demonstrated that EI-VOM combined with CA significantly increased the rate of bidirectional MI blocks compared with RFCA alone in AF patients (RR: 1.50, 95% CI: 1.34–1.67, *p* < 0.001). The duration for achieving a complete MI block was generally shorter in EI-VOM + CA group with fewer applications compared to the CA alone group [18,25,42]. However, the duration of fluoroscopy during the procedure may increase [18]. Although the adoption of EI-VOM as the primary treatment for all PMF cases remains contentious, it should be considered before resorting to epicardial ablation [21,78], especially in instances of resistant PMF, given its minimally invasive nature and reduced risk of complications.

## 7. Pre-Procedural Assessment of VOM

Assessing the anatomy of the VOM before catheter ablation procedures for AF can significantly reduce radiation exposure, contrast medium use, and the risk of complications. While visualizing the VOM with standard CT imaging is usually difficult, a specialized CT acquisition protocol has been developed and reported to improve visualization [79,80]. Takagi et al. [80], conducted a prospective study including 258 AF patients undergoing preprocedural contrast CT imaging. Initially, a standard protocol emphasizing peak LA enhancement was used for 132 patients; however, this was later adjusted to an optimized protocol targeting both the LA and CS for the remaining 126 patients. The optimized protocol required a higher volume of contrast media (90 mL vs. 70 mL) but resulted in similar radiation exposure (243 ± 138 mGy cm vs. 243 ± 86 mGy cm; *p* = 0.16).

In the optimized protocol, an initial bolus of 50 mL of pure (100%) iodine contrast medium was administered at a rate of 5 mL/s, followed by 40 mL at 3 mL/s, and finally 20 mL of pure (100%) saline at the same rate. This precise administration sequence ensures optimal contrast enhancement, facilitating clearer visualization of the vein of Marshall. The acquisition was timed to occur 20 s after detecting LA chamber enhancement (threshold of 100 Hounsfield units [HU]). Sublingual nitroglycerin was given before CT acquisition to enhance coronary venous flow [79,80].

In patients with a detectable VOM on venography, the vein was visualized in 38% of the conventional CT group and 65% of the optimized protocol group. Parameters such as the distance from the CS ostium to the VOM take-off (36 ± 7 mm), VOM diameter (1.6 ± 0.3 mm), and take-off location (superiorly in 68% and postero-superiorly in 32%) were useful for cannulation of the VOM. Despite the implementation of an optimized CT protocol, the VOM remains undetectable in approximately one-third of patients, especially in those with less epicardial fat around the CS. Nonetheless, integrating CT-derived VOM geometries into the 3D mapping system enables quicker and less invasive VOM catheterization, thus facilitating EI-VOM procedures.

## 8. Practical Approaches for EI-VOM

**Figure 5:**
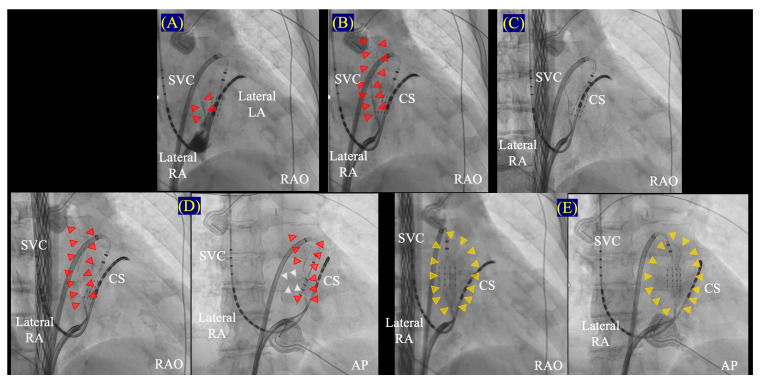
**EI-VOM with femoral approach.** Balloon occlusion of the CS ostium during venography enhances visualization of the VOM ostium (red triangles) with the Lumen BeeAT (Japan Life Line, Tokyo, Japan) inserted from the jugular vein (**A**). A 5 French Judkins Right (JR) catheter is wedged into the VOM ostium and slow contrast dye injection enables visualization of the distal branches (red triangles) (**B**). A 0.014-inch angioplasty wire is threaded through the 5Fr JR catheter, followed by an over-the-wire angioplasty balloon (6–8 mm in length, with a nominal diameter of 1.5–2.5 mm) for inflation (**C**). VOM distribution should be carefully assessed with different angles by VOM venography with balloon occlusion. The distribution area (red and white triangles) can be predicted and projected onto a 3D mapping system using a mapping catheter under fluoroscopy guidance. In this case, the AP view demonstrates the posterior distribution (white triangles) close to the esophagus, meaning that ablation in front of the esophagus can be avoided for left pulmonary vein isolation (**D**). After the confirmation of the VOM distribution with contrast, 96% ethanol (10 mL, 8.08 g, 808 mg/mL) is slowly injected at a rate of 0.5–3 mL per minute. Following injection, a selective venography of the VOM is conducted to check for vein dissection or perforation. This procedure is generally repeated until 6 to 10 mL of ethanol is administered, resulting in a stained area (yellow triangles) that reveals the impact (**E**). AP, anteroposterior; CS, coronary sinus; RA, right atrium; RAO, right anterior oblique view; SVC, superior vena cava.

With a solid understanding of fluoroscopic anatomy and the use of angioplasty tools, the VOM ethanol technique is relatively straightforward and achieves success in about 90% of cases [14,17,22,23,75]. Failure is more likely in cases where the VOM is absent or has a plexus-like distribution [30]. Although EI-VOM was initially demonstrated with a right internal jugular or left subclavian approach by Valderrábano M et al., we present a femoral approach here (Figure 5).

**Figure 6:**
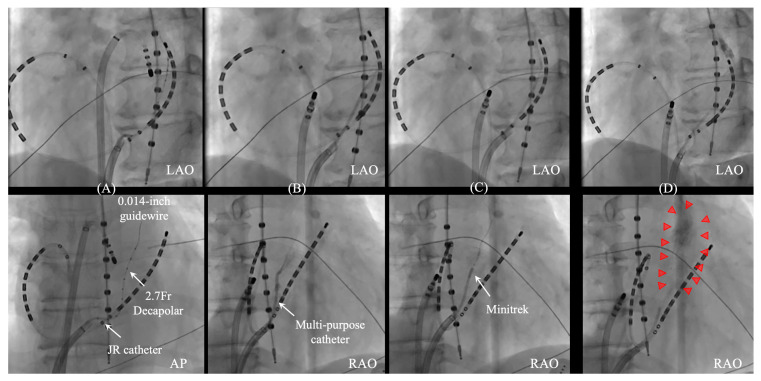
**Successful EI-VOM achieved by switching from a Judkins Right (JR) catheter to a multi-purpose catheter.** Initially, the 2.7 French decapolar catheter (EP-star FIX AIV, Japan Life Line, Tokyo, Japan) was used to record electrograms from the VOM, with a 0.014-inch guidewire running through the JR catheter (**A**). However, the JR catheter had difficulty engaging with the VOM ostium due to its unsuitable tip angle. Switching to a multi-purpose catheter allowed the catheter to properly engage with the VOM ostium (**B**). Following this, an over-the-wire angioplasty balloon was positioned under the guidance of the 0.014-inch guidewire and then inflated at the VOM ostium (**C**). This setup allowed for a successful ethanol infusion into the VOM (**D**). Successful performance with sufficient stain is observed (red triangles) (**D**). AP, anteroposterior; JR, Judkins Right; LAO, left anterior oblique view; RAO, right anterior oblique view.

(1) A long steerable sheath is introduced and maintained within the CS. To improve visualization of the ostium of the VOM during venography, a balloon occlusion at the CS ostium might be suggested if the VOM ostium is not easily distinguishable. It may be useful to place a catheter in the CS through the jugular vein as a landmark to identify the ostium of the VOM. The right anterior oblique (RAO) view is preferred to better discriminate the VOM ostium.

(2) As a sub-selector catheter, a 5Fr JR catheter can be introduced into the CS with or without a 0.35 wire, potentially wedging the VOM ostium. Typically, the VOM ostium branches just before the Vieussens valve, positioned at the 9–12 o’clock direction [80] in the RAO view. Depending on the angle of the VOM ostium, catheters like LIMA or multi-purpose catheters may be selected (Figure 6). While a 6Fr catheter used for angioplasty or a sheath designed for left ventricular pacing lead delivery (from the jugular vein) were originally used in the previous report [16,21] we performed the procedure with a 5Fr diagnostic catheter.

(3) Once engagement with the VOM is achieved, a 0.014-inch angioplasty wire (Whisper (Austin, TX, USA); Abbott (Chicago, IL, USA) or Sion Blue (Seto, Japan), Cruise (San Francisco, CA, USA), and Agosal (Seto, Japan); Asahi (Tokyo, Japan)) is inserted into the VOM, followed by advancing and inflating an over-the-wire angioplasty balloon (6–8 mm length, 1.5–2.5 mm nominal diameter, e.g., Minitrek, Abbott, USA). Wedging the JR catheter at the VOM ostium can facilitate this step, although cautious manipulation is necessary to avoid dissecting the VOM.

(4) After proper wire selection, the balloon is delivered to the VOM ostium, and selective VOM venography is performed by injecting contrast into the balloon lumen, revealing the VOM distribution.

(5) Following confirmation of balloon occlusion and VOM distribution through contrast injection, ethanol (96% ethanol, 10 mL, 8.08 gr, 808 mg/mL) is slowly injected at a rate of 0.5–3 mL over 1 min. Afterward, selective VOM venography is performed to assess the distribution of contrast dye and examine if the vein is dissected or perforated. This procedure is usually repeated until a total of 6 to 10 mL of ethanol is delivered.

Recently, a multipolar catheter with internal cardioversion capability (Lumen BeeAT, Japan Life Line, Tokyo, Japan) and the lumen for the 2.7Fr decapolar catheter (EP-star FIX AIV, Japan Life Line, Tokyo, Japan) have been available in Japan. Once the Lumen BeeAT is inserted in the CS, the VOM ostium can be selected by a 0.014-inch wire (Whisper; Abbott or Sion Blue, Cruise, and Agosal; Asahi) through the EPstar Fix AIV, which may work well for recording VOM electrograms. This catheter system covers the superior vena cava, right atrium, CS, and VOM simultaneously, allowing for efficient exploration of non-pulmonary vein foci after internal cardioversion.

## 9. Angiographic Assessment of the VOM and Prediction of the Impact of the EI-VOM

To prevent the incorrect injection of ethanol into the wrong vein, it is crucial to have a thorough understanding of the distribution between the CS and its branches. Valderrábano C et al. [30] outlined a consistent pattern that begins at the CS ostium. The LA veins include a septal vein, an inferior atrial vein, the VOM, veins from the LAA, and an anterior roof vein. Additionally, there are LA veins that are not connected to the CS, such as roof veins and posterior wall veins, often associated with extracardiac collaterals. Understanding this pattern can help ensure proper administration of ethanol during procedures involving the CS and its branches. The VOM is typically the most frequently identified left atrial vein, usually originating just proximal to the valve of Vieussens, 4.25 ± 2.57 cm from the CS ostium, with substantial variability. To ensure accurate localization of the VOM distribution and to predict the effects of EI-VOM, it is recommended to use a mapping catheter to trace the fluoroscopic VOM’s course and integrate it into a 3D map. This approach also helps avoid misinjecting ethanol into unintended locations, such as the LAA vein, which can occur if the VOM originates from a higher segment of the CS or is positioned closer to the CS ostium. Although the VOM is generally a true atrial vein, featuring branches and noticeable venules that drain the surrounding atrial tissue in 78%, a venous plexus was observed at the origin of the VOM in 10%, characterized by a lack of a clear dominant vein and instead the presence of a few or more branches. In 12%, the VOM was a stump with minimal or no atrial branches, showing a sudden transition from the CS into a dead-end structure or cul-de-sac. The VOM typically extends from the CS along the lateral ridge toward the left pulmonary veins. While it is visible up to the level of the LIPV in 72.8% of cases, it reaches the LSPV in only 9.6%. Additionally, in 17.6% of cases, the VOM does not even reach the LIPV.

Predicting lesion size and lesion distribution may sometimes be challenging in EI-VOM. The impact of ethanol-induced low voltage was assessed in 114 patients undergoing EI-VOM-based ablation for AF by Kamakura T et al. [81]. The two most frequently impacted segments were the inferior portion of the ridge (82.5%) and the first half of the mitral isthmus (pulmonary vein side) (92.1%). A residual gap on the mitral isthmus was more frequently observed on the annular side [20]. The lower left posterior wall next to the esophagus was affected by the EI-VOM in 19.3% of cases. This suggests that EI-VOM might be a safe and effective way to target arrhythmogenic structures near the esophagus in the posterior LA [82]. This could be useful in cases where RF ablation is difficult due to frequent temperature increases. Kamakura T et al. demonstrated that low-voltage extensions can be anticipated by the presence of visible anastomosis of the VOM with roof or posterior veins, but possibly with low predictive values [81]. Landra F et al. developed a way to measure the effect of EI-VOM in 42 AF patients with identifiable VOM [83]. Using fluorographic images processed with Weasis DICOM medical viewer software v4.02 (University Hospital Geneva, Switzerland), they created the myocardial staining index (MSI). The study found a strong link between the MSI and the expansion of low-voltage zones (r = 0.776; *p* = 0.001). Higher MSI scores were associated with shorter ablation times, fewer radiofrequency applications for the achievement of MI blocks, and successful MI blocks using only endocardial ablation. On the other hand, Takagi T et al. [76] evaluated, in 204 AF patients undergoing EI-VOM, the clinical significance of localized staining during EI-VOM, presumably caused by injury to VOM venules, and is visualized as leakage of contrast medium from a focal point of origin. The study found that localized staining occurred in 27% of patients, but it had no significant clinical effects, such as adverse outcomes or complications like pericardial effusion.

## 10. Complications of EI-VOM

The most commonly encountered complications were cardiac tamponade and pericardial effusion. A sub-analysis of a meta-analysis [45] comprising ten studies, pooling data from four specific studies [20,21,42,48], revealed no significant difference in these complications between groups treated with EI-VOM + CA and those with CA alone (cardiac tamponade: RR: 0.80, 95% CI: 0.29–2.19, *p* = 0.67; pericardial effusion: RR: 1.10, 95% CI: 0.48–2.48, *p* = 0.83). In the single-arm meta-analysis, cardiac tamponade occurred in seven studies [20,21,33,42,49,51], with nine cases observed in 644 patients (PR: 0.8%, 95% CI: 0.1–1.5%) who underwent EI-VOM combined with CA. Among five studies [26,42,48,49,51], there were 31 instances of pericardial effusion observed in 512 patients (PR: 4.0%, 95% CI: 0.7–7.3%).

**Figure 7:**
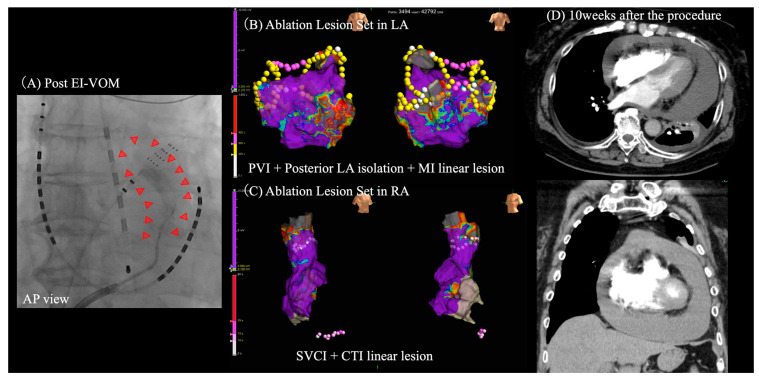
**An example case of delayed cardiac tamponade.** A 78-year-old female underwent EI-VOM (**A**), followed by PVI, MI line, and posterior LA isolation in the LA (**B**). Additionally, she underwent a CTI linear lesion and SVCI in the RA (**C**) to treat long-standing persistent AF. Initially, the outcomes were positive, with a significant removal of AF symptoms. During follow-up visits at 3 and 8 weeks post-procedure, the patient reported no specific symptoms, and her BNP level remained stable at <50 pg/mL. However, by the 9th week, she began experiencing shortness of breath, prompting her visit to the emergency department in the 10th week. On examination, she presented with mild hypotension (blood pressure 88/60) and sinus tachycardia (115 bpm). Her hemoglobin level remained consistent at 11 g/dL, but her BNP level had increased to 120 pg/mL. A CT scan revealed a large pericardial effusion (**D**). Subsequent pericardiocentesis drained 1100 mL of pale red fluid from the pericardial sac. AF, atrial fibrillation; AP, anteroposterior view; BNP, brain natriuretic peptide; CT, computed tomography; CTI, cavotricuspid isthmus; EI-VOM; ethanol infusion to the vein of Marshall; LA, left atrium; MI, mitral isthmus; PVI, pulmonary vein isolation; RA, right atrium; RAO, right anterior oblique view; SVCI, superior vena cava isolation.

Kamakura T et al. [14] reported an 88.9% success rate for EI-VOM in 634 out of 713 planned procedures. However, complications included 20 cases (2.8%) of VOM perforation, identified by iodine leakage into the pericardial space, and 13 cases (1.8%) of pericarditis, marked by chest pain and limited pericardial effusion. There were fourteen serious complications (2.0%) documented: seven cases (1.0%) of tamponade, with six delayed and necessitating pericardiocentesis days after the procedure (range: 7–106 days), four strokes (0.6%), one case of anaphylactic shock (0.1%), one atrioventricular block (0.1%), and one left appendage isolation (0.1%). Only four severe complications occurred during the procedure. Despite a low overall complication rate, delayed cardiac tamponade emerged as a notable post-EI-VOM complication (Figure 7). Another study involving 129 patients reported a relatively higher incidence of delayed cardiac tamponade at 3.1% [84]. While both sealed micro-perforations in the atrium and subacute pericarditis, like Dressler’s syndrome, may contribute to delayed cardiac tamponade, pericarditis appears to be more frequently linked to this complication following EI-VOM. This conclusion is drawn from a higher occurrence of pericarditis and delayed cardiac tamponade, along with predominantly serous rather than hemorrhagic pericardial effusion, contrasting with acute cardiac tamponade.

## 11. Conclusions

The LOM, which is insulated from the LA by fatty tissue, is an epicardial structure containing sympathetic nerves, veins, and muscular bundles that connect to the LA. The LOM can not only act as a trigger but also as a substrate of AF and AT. Additionally, the LOM can be a source of arrhythmogenic autonomic nerves, contributing to the development of these tachyarrhythmias. It is often involved in complex ATs, especially after endocardial ablation at the mitral isthmus. EI-VOM provides a safe and effective way to eliminate this arrhythmogenic structure.

## Data Availability

The datasets used and/or analyzed during the current study are available from the corresponding author upon reasonable request. However, some data in the present manuscript were used under license from Elsevier for the current review and are not publicly available from us.
